# Supplementary material for: A global meta-analysis of yield stability in organic and conservation agriculture
Source: Nat Commun. 2018 Sep 7;9:3632. doi: 10.1038/s41467-018-05956-1 (PMC6128901; doi:10.1038/s41467-018-05956-1)
Supplement: Supplementary file 1 — Supplementary Information [file 41467_2018_5956_MOESM1_ESM.pdf]

Supplementary Information

A global meta-analysis of yield stability in organic and conservation agriculture

by

Samuel Knapp and Marcel G.A van der Heijden

Supplementary Note 1

It has previously been observed, that the standard deviation is often correlated to the mean. This relationship, known as Taylor's power law [1], has been shown to also hold true for crop yields when comparing the natural log of the variance and the natural log of the mean [2]. The coefficient of variation (CV) is used as one approach to correct for this positive relationship of the standard deviation and the mean. Krebs [3] noted that the use of CV is only appropriate, when the slope of the log(SD) on log(mean) is equal to 1 (corresponding to a slope of 2 in the more commonly used regression of log(variance) on log(mean)). When looking at observed mean yields and the standard deviation across observations (not ratios), a significant positive correlation can be observed between log(SD) and log (mean), which results in a negative slope of log(CV) on log(mean) in both datasets (Supplementary Fig. 4). This indicates, according to Krebs [3], that the CV cannot be used to correct for the positive relationship between standard deviation and the mean. However, in our analysis we did not compare the variability between observations, but between treatments, as we assessed the ratio between treatments. We therefore regressed log(SD) and log(CV), respectively, on log(mean) of both treatments within each observation. As this regression only used two points (both treatments) from each observation, the estimated slopes showed a wide and non-symmetric distribution (Supplementary Fig. 5) and we therefore used the pseudo-median (estimated by the Hodges-Lehmann estimator) instead of the mean as location parameter. We calculated 95% confidence intervals and tested against the alternative hypothesis of independence of log(SD) from log(mean), i.e. the true location parameter is not equal to 0 for the regression of log(SD) and -1 for the regression of log(CV) using the wilcox.test() function in R. The estimated overall slopes of log(SD) on log(mean) ($b=-0.42$ and $b=-0.09$ for the organic and no-tillage dataset, respectively), were not significantly ($\alpha=0.05$) different from zero ($P=0.06$ and $P=0.65$ for the organic and no-tillage dataset, respectively). This indicates that the SD was independent of the mean, which is also supported by our finding that there is no difference in absolute stability. Furthermore, the

slope of log(CV) on log(mean) were, on average, b=-1.42 and b=-1.09 for the organic and no-tillage dataset, respectively, and not significantly (alpha=0.05) different from -1 (P=0.06 and P=0.65 for the organic and no-tillage dataset, respectively), indicating that the CV is inversely related to the mean. This is further confirmed by the regression of the ratios of the stability measures on the mean yield ratios (see Fig. 6 in the main text). It is important to note, that the significance level for both regressions (log(SD) and log(CV) in the organic dataset is only very slightly above the threshold of alpha=0.05, indicating that there is some negative relation between log(SD) and log(mean). This would mean that conventional agriculture has not only higher mean yield, but also increased absolute stability. However, the actual meta-analysis procedure does not indicate this (Fig. 1 in the main text).

The observed independence (or even negative relationship) of log(SD) and log(mean) does not indicate that Taylor's Power Law occurred. It is therefore important to carefully interpret the difference in variation measured by CV, as the difference is mainly due to the difference in variation measured by SD, which is indicated by the linear negative relationship between log(CV) and log(mean). Furthermore, the positive relationship between SD and mean across observations does not influence our results as we used pairwise comparisons between treatments within observations by using the ratios of the mean yield or the respective stability measures.

Supplementary Figures

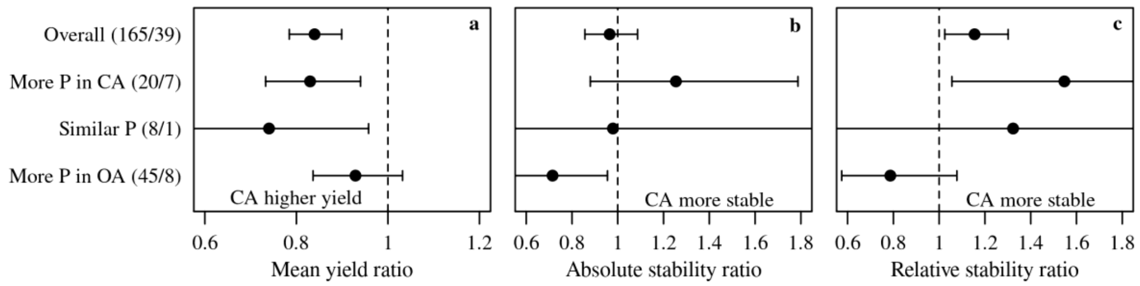

Supplementary Fig. 1: Effect of phosphorus input on yield and yield stability comparing organic and conventional agriculture. ***a*** *Mean yield ratio,* ***b*** *Absolute stability ratio,* ***c*** *Relative stability ratio for organic (OA) versus conventional (CA) agriculture for different levels of phosphorus (P) input. Numbers in parentheses denote the number of observations and studies. A ratio of 1 means that there is no difference between organic and conventional managed systems while values <1 indicate higher yield for conventional agriculture. For both stability measures ratios >1 indicate greater absolute and relative stability for conventional agriculture. Values are mean effect sizes with 95% confidential intervals. Yield or stability were deemed significantly different between organic and conventional agriculture if the 95% confidential intervals of the ratios did not overlap one.*

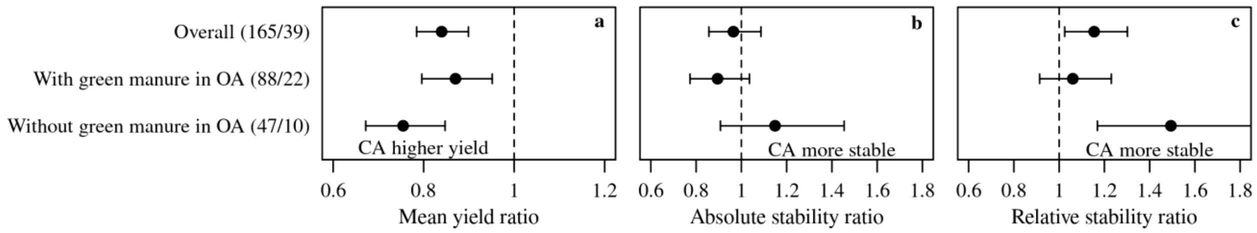

Supplementary Fig. 2: Effect of green manure on yield and yield stability comparing organic and conventional agriculture. ***a*** *Mean yield ratio,* ***b*** *Absolute stability ratio,* ***c*** *Relative stability ratio of organic (OA) versus conventional (CA) agriculture using all observations (Overall) and observations with or without the application of green manure in organic agriculture. Numbers in parentheses denote the number of observations and studies. A ratio of 1 means that there is no difference between organic and conventionally managed systems while values <1 indicate higher yield for conventional agriculture. For both stability measures, ratios >1 indicate greater absolute and relative stability for conventional agriculture. Values are mean effect sizes with 95% confidential intervals. Yield or stability were deemed significantly different between organic and conventional agriculture if the 95% confidential intervals of the ratios did not overlap one.*

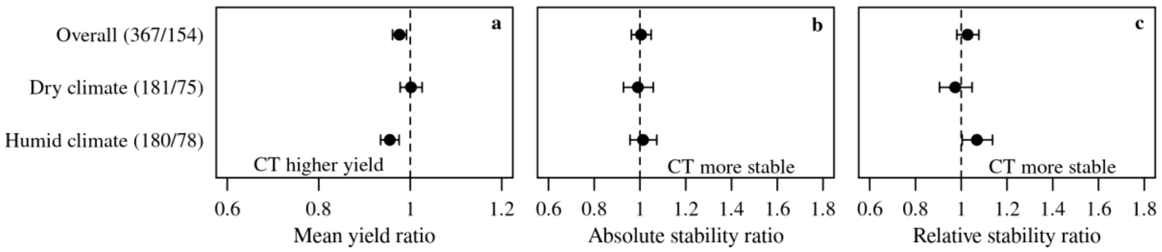

Supplementary Fig. 3: Effect of climate on yield and yield stability comparing no-tillage and conventional tillage. ***a*** *Mean yield ratio,* ***b*** *Absolute stability ratio,* ***c*** *Relative stability ratio of no-tillage (NT) versus conventional tillage (CT) for dry and humid climates. Following Pittelkow et al.* [4] *we defined dry climates based on the aridity index with values less than 0.65. Numbers in parentheses denote the number of observations and studies. A ratio of 1 means that there is no difference between no-tillage and conventional tillage while yield values <1 indicate higher yield for conventional tillage. For both stability measures values >1 indicate greater stability for conventional tillage. Values are mean effect sizes with 95% confidential intervals. Yield or stability were deemed significantly different between no-tillage and conventional tillage if the 95% confidential intervals of the ratios did not overlap one.*

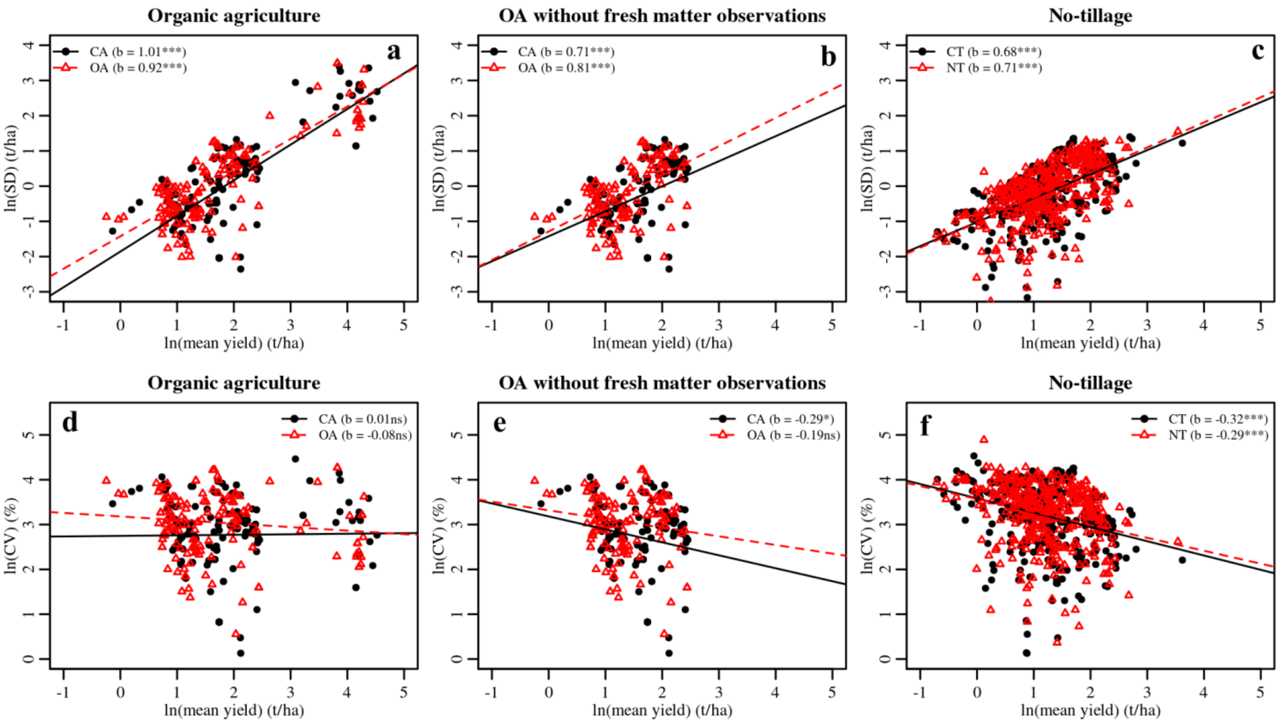

Supplementary Fig. 4: Relationship of observed mean yield and measures of variation. ***a-c*** *Relationship of observed mean yield and observed standard deviation,* ***d-e*** *Relationship of observed mean yield and coefficient of variation for the dataset comparing organic (OA) and conventional agriculture (CA) (****a****,* ***b****,* ***d****,* ***e****) and the dataset comparing no-tillage (NT) and conventional tillage (CT) (****c****,* ***f****), respectively. Each dot represents one multiple year observation (MYO) and both axes are on the ln scale. The regression line was fitted separately for each treatment on ln-transformed values, i.e. ln(y) = a + b * ln(x), where y was the respective stability measure (standard deviation or coefficient of variation) and x was the mean yield ratio. The dataset comparing organic and conventional agriculture contained several observations with a mean yield greater than 20 t/ha (****a****,* ***d****). These are observations for fruits or vegetables, where yield was determined on fresh matter. To account for that, the relationship is also shown after these observations have been removed (****b****,* ***e****). * and *** denote significance at P<0.05 and P<0.001, respectively, for a t-test with H0: b=0, and n.s. denotes non-significant (P>0.05).*

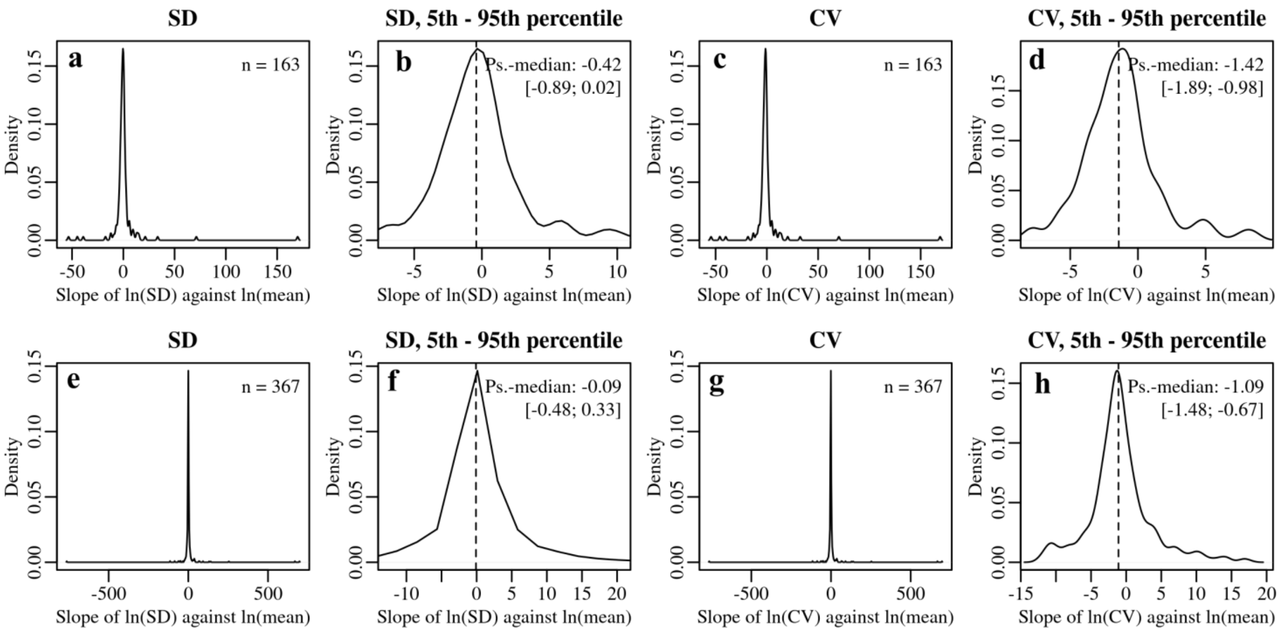

Supplementary Fig. 5: Relationship of observed mean yield and measures of variation within each observation. Kernel density plots of the distribution of the slope b from the regression of log(SD) and log(CV), respectively, on log(mean), within each observation for the dataset on organic agriculture (**a**-**d**) and no-tillage (**e-h**). In the dataset on organic agriculture, two MYOs had exactly the same yield for both treatments, and it was thus not possible to calculate the regression for these two MYOs. In order to better visualize the distribution, the distribution between the 5th and 95th percentile is shown additionally (**b**, **d**, **f**, **h**). The dashed line indicates the pseudo-mean [95% confidence intervals] (estimated by the Hodges-Lehmann estimator).

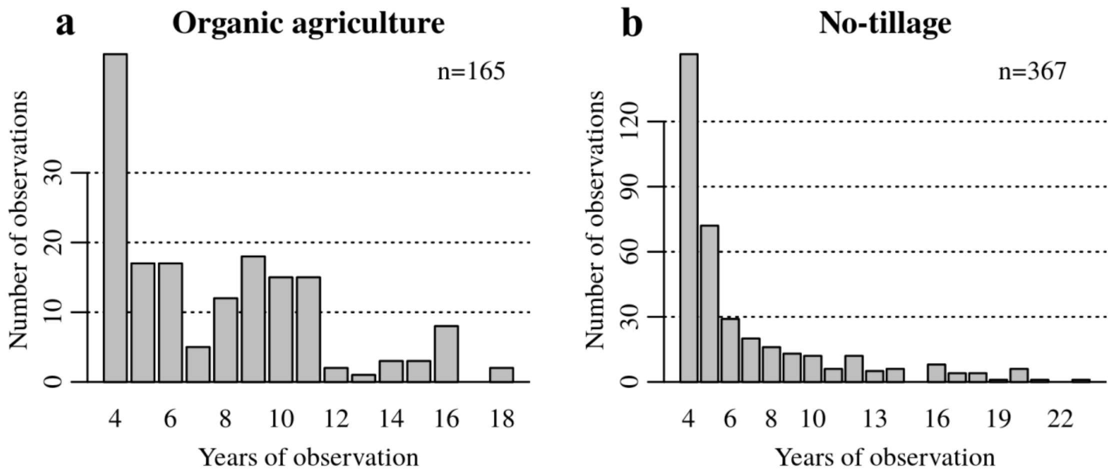

*Supplementary Fig. 6: Number of years of observation. Number of multiple year observations in relation to the number of years of observation in the dataset on organic agriculture (**a**) and on no-tillage (**b**). In the dataset on organic agriculture the observations are spread more equally over the whole range, whereas for the dataset on no-tillage 60% of all observations are based on 4 or 5 years of observation.*

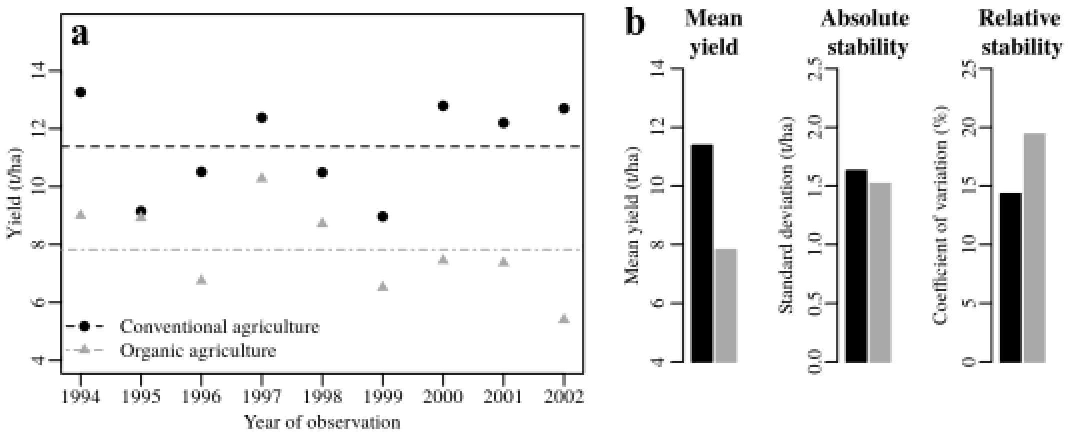

Supplementary Fig. 7: Example of a study monitoring the effects of organic and conventional agriculture on maize yield from 1994 to 2002. ***a*** *The actual yield for each year (1994-2002).* ***b*** *Mean yield, absolute stability and relative stability (i.e. the coefficient of variation, which measures the temporal variability across years relative to the mean yield level across those years). This study provided one multiple year observation as input for the meta-analysis. Data obtained from Denison et al.* [5].

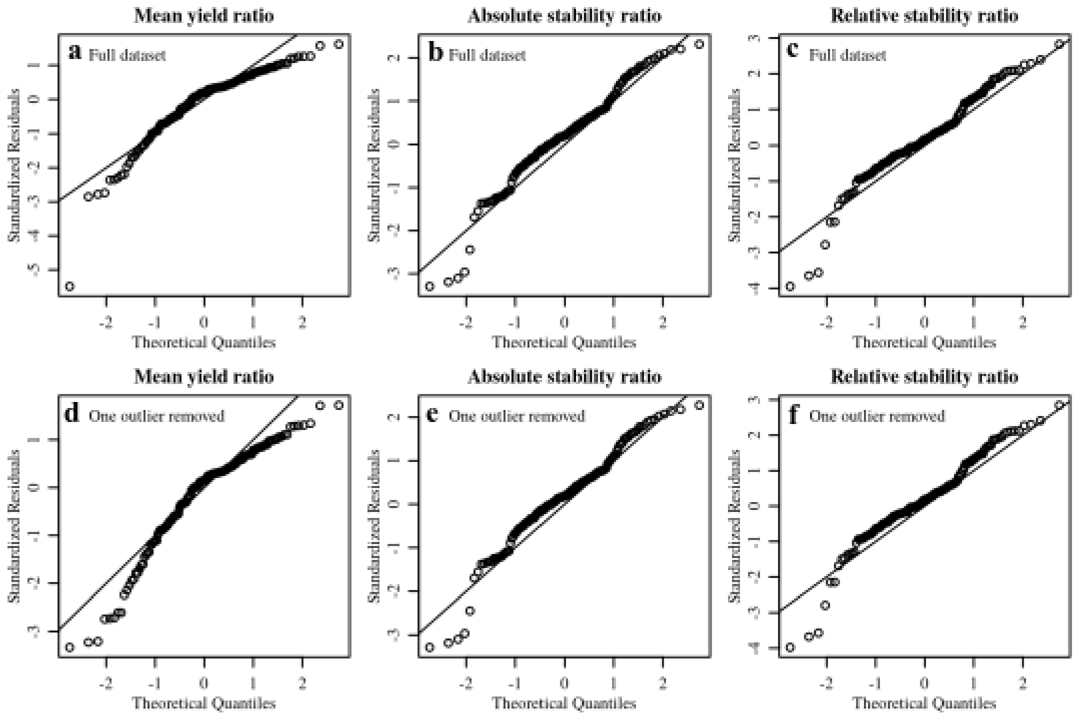

Supplementary Fig. 8: QQ-plots of the standardized residuals from the overall model for the dataset on organic farming. ***a**-**c*** *Distributions including the outlier.* ***d-f*** *Distributions after one outlier was removed (see methods).*

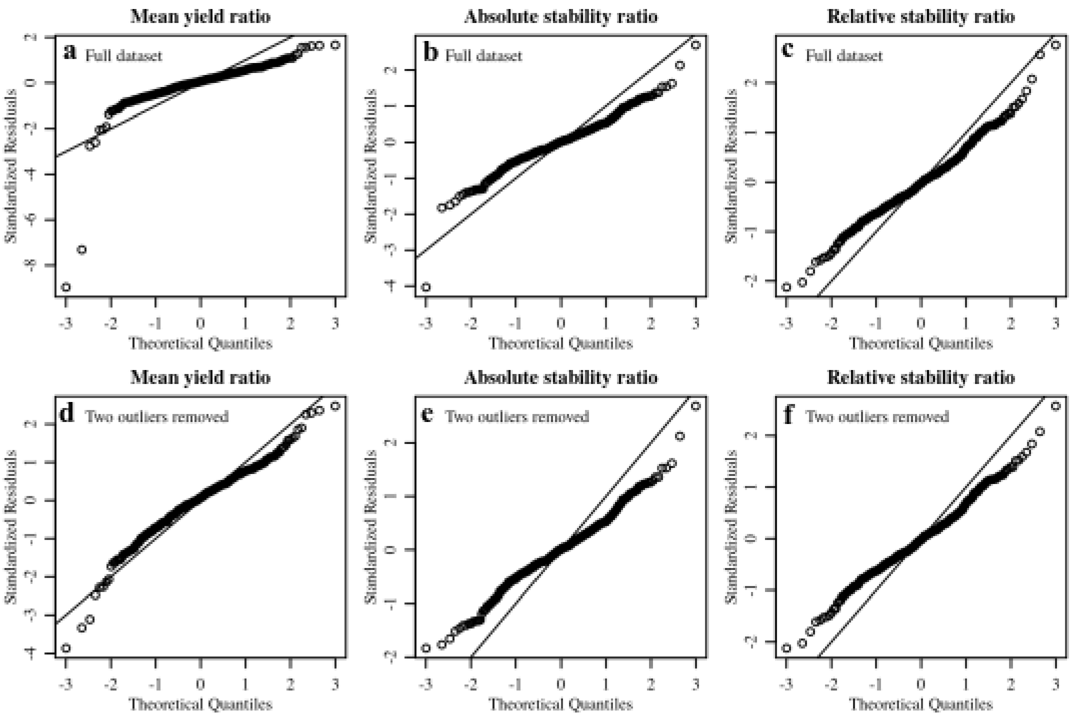

Supplementary Fig. 9: QQ-plots of the standardized residuals from the overall model for the dataset on no-tillage. ***a-c*** *Distributions including the outlier.* ***d-f*** *Distributions after two outliers were removed (see methods).*

Supplementary Tables

Supplementary Table 1: Estimates for mean yield, absolute and relative stability for all species contained in the dataset on organic agriculture [6].

Crop species	Observations/ Studies	Mean yield ratio			Absolute stabilty ratio			Relative stabilty ratio		
		Mean	lower CL	upper CL	Mean	lower CL	upper CL	Mean	lower CL	upper CL
**maize**	45/18	0.87	0.80	0.95	0.96	0.79	1.17	1.12	0.91	1.37
**soybean**	34/13	0.85	0.78	0.93	1.34	1.08	1.66	1.56	1.25	1.96
**wheat**	29/10	0.73	0.66	0.81	0.78	0.59	1.03	1.09	0.81	1.45
**oat**	10/4	0.88	0.74	1.06	1.05	0.76	1.45	1.15	0.79	1.66
**tomato**	9/4	1.01	0.86	1.19	0.52	0.34	0.79	0.51	0.33	0.80
**barley**	11/3	0.67	0.57	0.79	1.11	0.73	1.67	1.60	1.03	2.47
**apple**	3/2	1.00	0.69	1.46	0.82	0.36	1.87	0.77	0.30	1.97
**alfalfa**	4/1	1.02	0.85	1.24	0.89	0.56	1.40	0.88	0.53	1.44
**beetroot**	2/1	1.03	0.74	1.45	1.83	0.57	5.88	1.77	0.54	5.78
**carrot**	2/1	1.06	0.75	1.51	1.27	0.40	4.08	1.19	0.36	3.90
**lettuce**	2/1	0.91	0.56	1.46	2.14	0.74	6.17	2.36	0.77	7.28
**potato**	2/1	1.00	0.65	1.54	0.77	0.24	2.48	0.77	0.23	2.58
**rye**	2/1	0.75	0.52	1.10	1.13	0.39	3.25	1.49	0.50	4.44
**spring wheat**	2/1	0.74	0.50	1.09	1.69	0.61	4.68	2.46	0.85	7.17
**bean**	1/1	0.65	0.27	1.57	0.32	0.11	0.90	0.50	0.13	1.94
**chard**	1/1	1.02	0.65	1.62	0.66	0.23	1.89	0.67	0.22	2.06
**cotton**	1/1	1.11	0.60	2.06	1.39	0.38	5.12	1.25	0.30	5.17
**elephant foot yam**	1/1	1.19	0.73	1.92	1.08	0.33	3.50	0.91	0.26	3.14
**flax**	1/1	0.56	0.33	0.95	0.66	0.27	1.59	1.18	0.44	3.18
**grapes**	1/1	0.91	0.54	1.55	0.95	0.35	2.55	1.04	0.35	3.09
**pumpkin**	1/1	1.18	0.60	2.33	0.92	0.32	2.60	0.80	0.23	2.75
**safflower**	1/1	0.87	0.53	1.41	0.81	0.34	1.96	0.93	0.35	2.46

Supplementary Table 2: Estimates for mean yield, absolute and relative stability for all species contained in the dataset on no-tillage [4].

Crop species	Observations/ Studies	Mean yield ratio			Absolute stabilty ratio			Relative stabilty ratio		
		Mean	lower CL	upper CL	Mean	lower CL	upper CL	Mean	lower CL	upper CL
**barley**	28/17	0.96	0.91	1.02	1.03	0.88	1.20	1.07	0.90	1.27
**cotton**	13/6	0.95	0.88	1.02	1.05	0.78	1.41	1.04	0.75	1.43
**maize**	108/55	0.95	0.93	0.98	1.01	0.93	1.09	1.07	0.98	1.16
**rice**	10/7	0.94	0.90	0.98	0.94	0.68	1.28	0.99	0.72	1.36
**sorghum**	15/12	1.04	0.96	1.13	1.04	0.85	1.27	0.96	0.78	1.20
**soybean**	53/31	1.01	0.98	1.04	1.01	0.91	1.12	1.02	0.91	1.14
**wheat**	114/65	0.99	0.97	1.01	1.01	0.93	1.09	1.03	0.94	1.12
**pea**	5/3	1.03	0.85	1.25	1.10	0.68	1.76	1.01	0.60	1.71
**bean**	4/4	1.04	0.85	1.27	0.86	0.62	1.19	0.75	0.50	1.12
**canola**	3/3	1.07	0.85	1.35	1.09	0.56	2.09	1.02	0.51	2.05
**oat**	3/1	1.12	0.87	1.44	0.82	0.51	1.30	0.75	0.44	1.27
**chickpea**	2/2	0.98	0.72	1.34	1.08	0.66	1.76	1.11	0.62	1.99
**sunflower**	2/2	1.14	0.79	1.65	0.94	0.45	1.97	0.79	0.33	1.88
**lentil**	2/1	1.17	0.86	1.59	0.88	0.55	1.39	0.76	0.44	1.32
**lupin**	2/1	1.07	0.81	1.41	0.97	0.66	1.42	0.90	0.56	1.44
**rye**	2/1	0.88	0.70	1.11	1.10	0.49	2.45	1.25	0.54	2.87

Supplementary Table 3: Corrections that were applied to the original dataset from Ponisio et al. [6]

Comparison	Column name	Original value	New Value	Reasoning
43	X_coord	-95.8	95.48	same value as other comparisons from the same study
349	Legume	non-legumes	non-legume	all other entries are are without plural s
349	Rotation	org	more org	to achieve similarity to other entries
476-478	Var ac. Years	yes	no	in year-org and year-con only one year, thus variance across years is not possible
919-920	Var ac. Years	yes	no	in year-org and year-con only one year, thus variance across years is not possible
1037-1039	year-org	2001;2002;2004	2001 to 2003	same format as other observations from same study
1037-1039	year-con	2001;2002;2004	2001 to 2003	same format as other observations from same study
961	year-org	1990 and 1992	1990 to 1991	same format as other observations from same study
961	year-con	1990 and 1992	1990 to 1991	same format as other observations from same study
941-953	Yield unit	Mg/ha	kg/ha	wrong unit, see original publication
220-264	Yield unit	kg/ha	Mg/ha	all maize entries had wrong unit, see original publication
62-65			completely removed	extracted values do not correspond to original publication, also only 3 years in original publication

Supplementary Table 4: Example of the generation of multiple year observation (MYOs) from single-year observations for selected comparisons in the dataset on organic agriculture. The column "study" and "comparison" refer to the original dataset by Ponisio et al. [6]

Study	Comp-arison	Author (year)	Year of obser-vation	Treatment		Yield		Multiple year observation (MYO)	Mean over years		SD over years		CV over years	
				conv	org	conv	org		conv	org	conv	org	conv	org
3	43	Archer et al. (2007)	2003	conv	org	3.7	2.73	A	3.57	2.81	0.31	0.49	11.6	5.7
3	8	Archer et al. (2007)	2004	conv	org	3.73	3.51							
3	9	Archer et al. (2007)	2005	conv	org	3.75	2.62							
3	10	Archer et al. (2007)	2006	conv	org	3.11	2.38							
27	302	Doltra et al. (2010)	2005	CF+C+F	OF+C+M	5.68	5.19	B	5.47	5.04	0.62	0.27	8.8	18.9
27	269	Doltra et al. (2010)	2006	CF+C+F	OF+C+M	5.84	5.14							
27	267	Doltra et al. (2010)	2007	CF+C+F	OF+C+M	4.54	4.64							
27	271	Doltra et al. (2010)	2008	CF+C+F	OF+C+M	5.81	5.18							
27	270	Doltra et al. (2010)	2005	CF+C+F	OF-C+M	5.68	4.92	C	5.47	3.95	0.62	0.84	8.8	4.7
27	282	Doltra et al. (2010)	2006	CF+C+F	OF-C+M	5.84	4.38							
27	268	Doltra et al. (2010)	2007	CF+C+F	OF-C+M	4.54	3.2							
27	275	Doltra et al. (2010)	2008	CF+C+F	OF-C+M	5.81	3.29							

Supplementary Table 5: Example of splitting multiple year observations (MYO) that contained subtreatments in the dataset on no-tillage from Pittelkow et al. [4]. MYO A did not contain any subtreatments. MYO B and C were listed in subsequent order in the original dataset, and MYO D and E were listed in alternating order.

Study	duration	subtreatments	order	MYO
Aase et al. (1997)	1	no		A
Aase et al. (1997)	2	no		A
Aase et al. (1997)	3	no		A
Aase et al. (1997)	4	no		A
Dalal et al. (2013)	6	yes	subsequent	B
Dalal et al. (2013)	7	yes	subsequent	B
Dalal et al. (2013)	8	yes	subsequent	B
Dalal et al. (2013)	9	yes	subsequent	B
Dalal et al. (2013)	10	yes	subsequent	B
Dalal et al. (2013)	6	yes	subsequent	C
Dalal et al. (2013)	7	yes	subsequent	C
Dalal et al. (2013)	8	yes	subsequent	C
Dalal et al. (2013)	9	yes	subsequent	C
Dalal et al. (2013)	10	yes	subsequent	C
Maurya (1986)	1	yes	alternating	D
Maurya (1986)	1	yes	alternating	E
Maurya (1986)	2	yes	alternating	D
Maurya (1986)	2	yes	alternating	E
Maurya (1986)	3	yes	alternating	D
Maurya (1986)	3	yes	alternating	E
Maurya (1986)	4	yes	alternating	D
Maurya (1986)	4	yes	alternating	E

7

Supplementary References

1. Nakagawa, S. et al. Meta-analysis of variation: ecological and evolutionary applications and beyond. Methods Ecol Evol 6, 143–152 (2015).
2. Lajeunesse, M. J. On the meta-analysis of response ratios for studies with correlated and multi-group designs. Ecology 92, 2049–2055 (2011).
3. Pittelkow, C. M. et al. Productivity limits and potentials of the principles of conservation agriculture. Nature 517, 365–368 (2015).
4. Taylor, L. R. Aggregation, Variance and the Mean. Nature 189, 732 (1961).
5. Döring, T. F., Knapp, S. & Cohen, J. E. Taylor's power law and the stability of crop yields. Field Crops Research 183, 294–302 (2015).
6. Krebs, C. J. Ecological Methodology. (Addison Wesley, 1998).
7. Denison, R. F., Bryant, D. C. & Kearney, T. E. Crop yields over the first nine years of LTRAS, a long-term comparison of field crop systems in a Mediterranean climate. Field Crops Research 86, 267–277 (2004).
